# Further improvement of our metrics—will plan S affect them?

**DOI:** 10.1080/03009734.2019.1691688

**Published:** 2019-12-17

**Authors:** Arne Andersson, Daniel Espes, Joey Lau Börjesson

**Figure 1.**
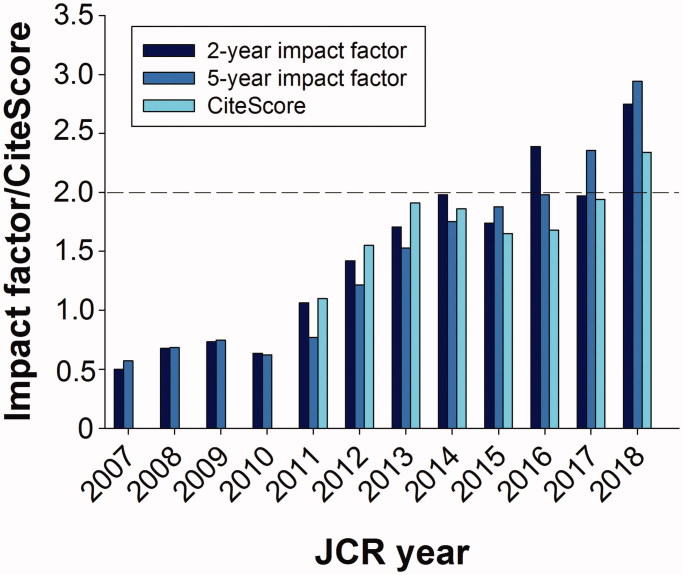
Impact factor or CiteScore of Upsala Journal of Medical Sciences.

Traditionally, the last issue—no. 4—of each volume of *UJMS* informs our readers about the performance of our journal in terms of metrics. It is a much-debated phenomenon (1), questioned by many researchers but loved by research administrators (2). As long as figures are pointing in the right direction, we tend to belong to the lovers. Thus, this time we can report new record figures for all three parameters of special interest—the 2- and 5-year impact figures (Clarivate) and the newly launched CiteScore (Elsevier). The 5-year score closed just below 3.0, and the most frequently used score—the 2-year value—a couple of tenths lower (Figure 1). We prefer to be judged by the former, not just because it gave us the highest score ever this year, but rather because small journals with few issues and articles are very sensitive to the fate of individual papers. A blockbuster can be very valuable, but its disappearance from the denominator after 2 years is equally detrimental. For *UJMS* the CiteScore figure, 2.34, is very close to these traditional impact factor (IF) scores. This is noteworthy, since most of the prestigious journals have a five-fold lower CiteScore value when compared with their IF figures (3). This is because of the fact that they all—and especially *New England Journal of Medicine*—have to include all published articles in the denominator of the CiteScore calculation.

**Figure 2.**
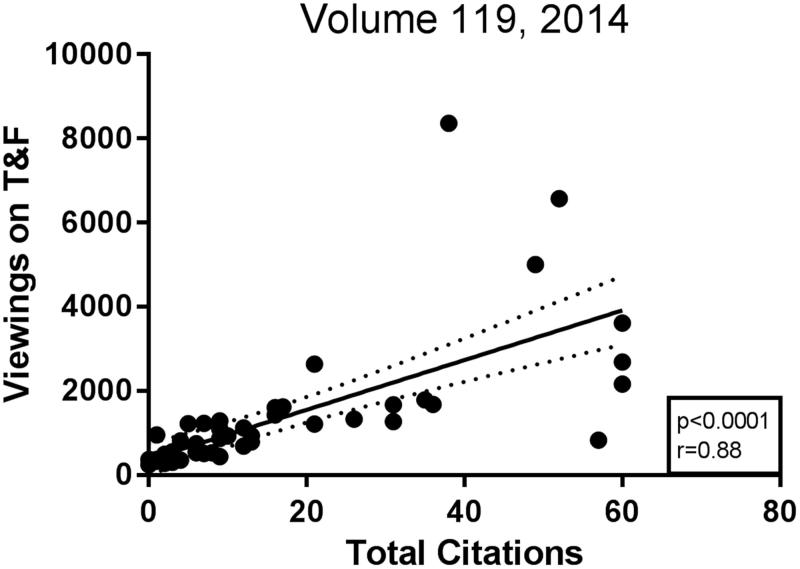
Viewings on Taylor & Francis correlate to total citations.

When estimating different metric values, the number of citations in recognised journals forms the basis for such calculations. Taking into account the electronic supervision of all activities at the journal’s website nowadays, it is possible to monitor all downloads of published articles and also see who the viewer is (4). One pertinent question, then, is to what extent there is a correlation between the viewings of an article and the number of citations of the same paper. Now and then you can hear the argument that articles should be judged by the numbers of readers—approximately the number of downloads on the journal’s website—rather than by the actual citation figures in different data bases. We therefore looked at these figures in one volume—volume 119, published in 2014—at a time point when both new citations and viewings had started to level off. Perhaps not surprisingly, there was quite a strong correlation between the numbers of citations and viewings (*r* = 0.88; *p* < 0.0001) (Figure 2). There are, however, quite a few extreme cases where many viewings do not necessarily predict a future as a top-score citation candidate. In the present volume of our journal, we have an example of one such article that was downloaded many times immediately upon its release in mid-April. Thus, we want to highlight the paper by Milutinovic et al. in issue 2, 2019, on surface glycans in prostasomes (5). Now, half a year later, no less than 1700 viewings have been registered. Will that paper become a citation classic? We would like to interpret this to indicate that this field of research, pioneered in a masterly way by our former editor Gunnar Ronquist, is becoming hotter and hotter (6). We encourage people active in this field to submit their papers to our journal. As always, we guarantee quick and reliable handling of manuscripts, as well as the open access strategy of our journal without APCs (article publication charges; see below).

The issues dwelt upon above are trivial when considering the big and most controversial subject in scholarly publishing these days—cOAlition S, or more frequently presented as ‘plan S’. When visiting their website, it says that plan S is ‘Making full and immediate open access a reality’ (7). The plan is supported by an international consortium of funders, and the task of cOAlition S is to take action towards the implementation of plan S. There are many well-known supporters of this initiative and prestigious funders like the EU, Wellcome, the Research Council of Norway, the Academy of Finland, and some Swedish funders such as Vinnova, Formas, and Forte. Interestingly, the Swedish Research Council has postponed their signing of the contract. When it was launched in September 2018, the start was planned for 2020, but this has now been postponed until 2021, because of lack of time for the implementation and some firm resistance amongst researchers.

What impact could these changes have for our journal? Quite likely, forcing researchers funded by public grants provided by national and European research councils to publish under open access policies would benefit our journal. *Upsala Journal of Medical Sciences* has applied gold Open Access for the last 10 years, i.e. all published articles have been fully accessible immediately upon release without a publication paywall. It is most likely that this feature of our journal has enhanced our performance substantially. When investigating the influence of the open access publishing format on the IF values of open access and closed access journals over a 5-year period, we found no obvious effect on the journals chosen for the review (4). In order to have a longer time perspective, we looked at the same journals 5 years later when the IF values for 2018 were released (Tables 1 and 2). Interestingly, there was no obvious increase of the values during this 10-year period. This was despite a remarkable increase of both the number of journals and articles. But, again, no differences between the two groups of journals were discernible. It is worthy of note that two Uppsala-based journals, one in the closed category (*Amyloid*; more than two-fold) and one in the open access (*UJMS*; almost four-fold) display quite obvious improvements. So, in essence we have nothing to fear. Open access publishing will most probably slowly increase, and fees will have to be paid in some way for the service publishers provide. APCs will become a more common phenomenon, and the level will depend on the quality and reputation characterising the journal. At present the board of our society has no plans for a change of our no-APC policy. Of course, such an offer attracts many contributions from research environments with meagre economic resources. It also constitutes a substantial work load for the editorial board. There is only room for some 40–50 published papers a year, and, with submission figures close to 300, there is a fairly high rejection rate. From the editors’ point of view, we have to attract more papers good enough for acceptance. And we take this opportunity to repeat that we offer a fast track for high-quality papers.

**Table 1.** Non-open access journals: impact factors.

Non-open access journals	IF 2009	IF 2018	Factor change
New England Journal of Medicine	47.050	70.670	1.50
The Lancet	30.758	59.102	1.92
Journal of Clinical Investigation	15.387	12.282	0.80
Diabetologia	6.551	7.113	1.09
Diabetes	8.585	7.199	0.84
Endocrinology	4.752	3.800	0.80
EMBO Journal	8.993	11.227	1.25
PNAS	9.432	9.580	1.02
Amyloid	2.115	4.919	2.33
Acta Oncologica	2.265	3.298	1.46

Mean factor change: 1.30 ± 0.16.

**Table 2.** Open access journals: impact factors.

Open access journals	IF 2009	IF 2018	Factor change
PLoS One	4.351	2.776	0.64
PLoS Medicine	13.050	11.048	0.85
PLoS Biology	12.916	8.386	0.65
BMC Biology	5.636	6.723	1.19
BMC Medicine	3.985	8.285	2.08
Virology Journal	2.435	2.464	1.01
Chinese Medical Journal	0.952	1.555	1.63
Swiss Medical Weekly	1.681	1.821	1.08
Journal of Translational Medicine	3.407	4.098	1.20
Upsala Journal of Medical Sciences	0.733	2.747	3.75

Mean factor change: 1.41 ± 0.30.

Let us finish this annual report with an apology. Perhaps some of you have noticed that the journal launched a new submission portal in May 2019. As editors of scholarly journals you would not notice this if it wasn’t for the sudden increase in your inbox of e-mails from prospective authors complaining about difficulties in crossing a veritable wall created by this new electronic manuscript central. It also quite soon became clear that resubmitting a revised manuscript involved many problems. The reasons for these shortcomings have not been resolved in detail as yet. Still, almost half a year later, there are complaints of the same nature, and the problems mainly involve the log-in process. We are struggling to return to the use of the old portal. Meanwhile, our advice is to either report problems to the technical support of Taylor & Francis or, alternatively, to contact the editor of the journal. It is impossible to assess how many authors have given up and sent their papers to other journals. Luckily, some manuscripts have nevertheless arrived at the editorial office, and submission figures are close to those of last year. Therefore, besides begging for your patience, we would like to hear from you if, or when, you face problems with your submissions. Press stop as of December 2, 2019; The old submission portal has now been reinstalled and, not surprisingly, the inconveniencies with the new version disappeared.
